# Examining the Factor Structure and Validity of the Depression Anxiety Stress Scale-21

**DOI:** 10.3390/ejihpe14110192

**Published:** 2024-11-20

**Authors:** Grant Jacobsen, Madeline P. Casanova, Alexandra Dluzniewski, Ashley J. Reeves, Russell T. Baker

**Affiliations:** 1 WWAMI Medical Education Program, University of Idaho, Moscow, ID 83843, USA; grant10@uw.edu (G.J.); mcasanova@uidaho.edu (M.P.C.); adluz@uidaho.edu (A.D.); 2 Idaho Office of Rural and Underserved Medical Research, University of Idaho, Moscow, ID 83843, USA; reev5522@vandals.uidaho.edu

**Keywords:** depression, stress, anxiety, confirmatory factor analysis, invariance analysis

## Abstract

Background: The prevalence of mental health disorders calls for valid and reliable instruments that are easy to administer and assess for clinicians and researchers. The Depression Anxiety Stress Scale-21 (DASS-21) is a commonly used instrument to assess psychological distress; however, model fit and internal reliability issues have been reported. Our objective was to assess the factorial and structural validity of the DASS-21. Methods: A confirmatory factor analysis (CFA) was conducted on the full sample (n = 1036) to assess the proposed three-factor DASS-21 using a priori cut-off values. Because model fit indices were not met, an exploratory factor analysis (EFA) was conducted to identify a parsimonious model. The resulting three-factor structure (i.e., DASS-9) was then assessed using CFA and multigroup invariance testing procedures. Results: The proposed three-factor DASS-21 did not meet model fit criteria. The DASS-9 did meet recommended model fit criteria and was invariant between sex, injury status, mental health diagnosis, and activity level groups. Statistically different group means were found between mental health diagnosis and activity level groups, while no differences between sex or injury status groups were found. Conclusions: The current study provides support to use a condensed DASS-21 instrument, such as the DASS-9. Future research is necessary to establish the DASS-9 prior to its adoption in research and clinical practice. Additionally, there is a need to identify and review all condensed versions of the DASS-21, so individuals know which instrument can be used for clinical or research purposes.

## 1. Introduction

Mental health disorders, such as depression and anxiety, significantly impact cognitive, emotional, and behavioral wellbeing [1]. Depression is the leading cause of disability globally, with an estimated 21 million adults in the United States alone reporting at a least one major depressive episode in 2020 [2]. Similarly, approximately 19.1% of adults in the United States have an anxiety disorder, equating to roughly 40 million people [3]. The toll of anxiety and depression often manifests in fatigue, muscle tension, difficulty sleeping, and irritability [3], while stress, a physiological reaction to an unfavorable stimulus, can also result in physical and emotional symptoms when the stressor becomes overwhelming [4]. Additionally, there is an increasing amount of evidence establishing relationships between mental health concerns, physical activity, and musculoskeletal injury [5,6]. For example, high levels of stress, depression, and anxiety have been linked to an increased risk of injury, and psychological conditions have also been found to significantly affect recovery outcomes [5,6,7,8]. This complex interplay between psychological health and physical recovery underscores the importance of including activity levels and injury status as variables when evaluating psychosocial distress. Given the prevalence and health toll of depression, stress, and anxiety, there is an escalating need for the development and validation of psychometrically sound instruments to measure psychological distress.

The Depression Anxiety Stress Scale-21 derived from the original DASS-42 [9] has emerged as a common instrument to assess the distinct symptoms of depression (e.g., dysphoria, hopelessness, devaluation of life, etc.), anxiety (e.g., autonomic arousal, skeletal muscle effects, etc.), and stress (e.g., difficulty relaxing, irritable/over-reactive, and impatient, etc.). The goal was to develop an easily administered instrument for clinical use, facilitating healthcare providers in addressing a significant public health issue. The DASS-21 has the potential to expedite assessment without compromising depth and to identify patients suffering from psychological distress [9].

Despite its wide use, the examination of its psychometric properties and factor structure yielded inconsistent results [10,11,12,13,14,15,16,17]. For example, a confirmatory factor analysis (CFA) on a three-factor (i.e., depression, stress, anxiety) correlated model and a bifactor model in nonclinical adults (i.e., adults without a mental health diagnosis) found poor fit for the three-factor model (CFI = 0.846; RMSEA = 0.074) but better fit for the bifactor model (CFI = 0.941; RMSEA = 0.05) [12]. In contrast, the oblique, three-factor model met some model fit indices (CFI = 0.945; TLI = 0.938; RMSEA = 0.042), while a single-factor model did not (CFI = 0.830; TLI = 0.812; and RMSEA = 0.072) in an undergraduate student sample [13]. A three-factor model in a sample of Portuguese students met some but not all model fit indices (CFI = 0.931; RMSEA = 0.038) [15], while model fit indices were not met in a Jordanian sample (CFI = 0.900; RMSEA = 0.072) [16] or in a sample of Persian healthcare professionals (CFI = 0.920; TLI = 0.910; RMSEA = 0.078) [17].

Researchers have also assessed the internal consistencies of the DASS-21 subscales, and like the model fit indices findings, internal consistency has often fallen outside of the recommended values (i.e., ≥0.70 and ≤0.89) [18,19]. Henry and Crawford [12] reported Cronbach’s α values of 0.88 (depression), 0.82 (anxiety), and 0.933 (stress); whereas, Osman et al. [13] found α values of 0.85 (depression), 0.81 (anxiety), and 0.90 (stress). Bottesi et al. [11] reported Cronbach’s α coefficients of 0.82 (depression), 0.74 (anxiety), and 0.85 (stress), while Thapa et al. [14] reported coefficients of 0.93 (depression), 0.79 (anxiety), and 0.91 (stress). Additionally, Laranieriera et al. [15] reported coefficients of 0.92 (depression), 0.90 (anxiety), and 0.92 (stress), Al-Dassean and Murad [16] reported coefficients of 0.84 (depression), 0.83 (anxiety), and 0.85 (stress), while Kakemam et al. [17] reported coefficients of 0.90 (depression), 0.87 (anxiety), and 0.89 (stress).

Given the inconsistencies in factor structure and internal consistency, the lack of sound model fit indices, and the lack of established measurement invariance in relevant subgroups, the purpose of this study was to test the model structure, internal consistency, and if appropriate, multigroup measurement invariance of the DASS-21 in a large sample of individuals. Because a psychometrically sound model was not found, the secondary purpose was to conduct an exploratory factor analysis to identify a parsimonious scale. The new model was then assessed using a covariance model and tested in subgroups of interest (i.e., sex, presence of a mental health diagnosis, physical activity level, injury status) using multigroup analysis procedures.

## 2. Methods

The University Institutional Review Board certified the study exempt. An electronic survey was developed using Qualtrics software (Qualtrics Inc., Provo, UT, USA, October 2023) that included the DASS-21 and a participant demographic questionnaire. Participants (i.e., emerging adults and adult participants) [20] were recruited using a combination of snowball and convenience sampling methods [21]. Researchers sent the link to personal contacts and advertised the study on social media pages. Additionally, study participants were recruited on the volunteer platform, ResearchMatch [22]. Participants provided informed consent prior to completing the survey.

### 2.1. Participants

A total of 1264 individuals completed the DASS-21 survey; 67 individuals reported scores that indicated univariate outliers (z scores ≥ 3.4), while an additional 161 reported scores that indicated multivariate outliers (Mahalanobis distance ≥ 38.93). These 228 respondents were removed from the dataset, leaving a total of 1036 for analysis. Respondents were aged 18–95 (mean age = 41.08 ± 16.67), with females accounting for 77.3% (n = 801) and males accounting for 21.7% (n = 225) of the sample. Participants were grouped by sex, injury status, mental health diagnosis, education level, and activity level (Table 1). Classification groupings for injury status and activity levels are presented in Supplemental Table S1.

### 2.2. Instrumentation

#### 2.2.1. Depression Anxiety Stress Scale-21

The DASS-21 has three distinct factors proposed to measure depression (e.g., I couldn’t seem to experience any positive feeling at all), anxiety (e.g., I experienced breathing difficulty), and stress (e.g., I found it hard to wind down) [9]. Participants rate how much the statement applied to them over the past week using a 4-point Likert scale (0 = Did not apply to me at all; 1 = Applied to me to some degree or some of the time; 2 = Applied to me a considerable degree or a good part of the time; 3 = Applied to me very much or most of the time), with higher scores indicating higher psychological distress.

#### 2.2.2. Participant Demographic Questionnaire

Participants completed a demographic questionnaire that included age, ethnicity, sex, education level, injury status, physical activity level, and previous or current diagnosis of a mental illness.

### 2.3. Data Cleaning

Qualtrics survey data were exported to the Statistical Package for Social Sciences Version 26 (SPSS, Inc., Chicago, IL, USA) and Analysis of Moment Structure (AMOS, SPSS, Inc.) Version 27 for analysis. Data normality was assessed using histograms and skewness and kurtosis values. Univariate and multivariate outliers were assessed using z-scores (cut-off value of |3.3| and Malahanobois distance (*p*-value = 0.01). A CFA was conducted on the full sample. Because model fit indices were not met [23], the full sample was randomly split into two equal samples (n1, n2; 518 responses per sample) that were similar across variables of interest. Sample n1 was used to conduct an exploratory factor analysis (EFA) to identify a more parsimonious solution. The EFA solution identified was then tested using a covariance model approach [23] in sample n2. The solution identified and tested in a covariance model then underwent multigroup (i.e., sex, activity level, injury status, history of mental illness) invariance testing.

### 2.4. Data Analysis Plan

#### 2.4.1. Confirmatory Factor Analysis

Analysis of Moment Structures Version 26 (AMOS, IBM, SPSS, Chicago, IL, USA) software was used to conduct a CFA with maximum likelihood extraction on the 3-factor, 21-item DASS-21. The following overall goodness of fit indices were evaluated, and model fit deemed acceptable if contemporary criteria were met: comparative fit index (CFI ≥ 0.95); Tucker–Lewis index (TLI ≥ 0.95); root mean square error of approximation (RMSEA ≤ 0.06); and Bollen’s incremental fit index (IFI ≥ 0.95) [23,24]. The likelihood ratio statistic (Chi-square) was also assessed but not used as a primary assessment of fit, because it is influenced by sample size variations [23,24]. In addition to overall model fit indices, localized areas of strain and parameter estimates (i.e., factor variances, covariances, indicator errors) were also evaluated [25].

#### 2.4.2. Exploratory Factor Analysis

An EFA using maximum likelihood extraction and direct oblimin rotation was conducted. Barlett’s test for sphericity (<0.001) and Kaiser–Meyer–Olkin measure of sampling adequacy (≥0.70) were assessed [18]. The following criteria were used to determine the appropriate number of factors to retain: (1) factors with eigenvalues > 1.0; (2) examination of the scree plot inflection point; (3) parallel analysis, which compares eigenvalues in the original dataset to a randomly ordered dataset [26]; and (4) factors that accounted for ≥5% of the variance [11,21,23]. Following extraction, items were assessed and removed individually using the following recommendations: loading < 0.40; cross-loading ≥ 0.30; low internal consistency; high bivariate correlations with another item; and theoretical or conceptual misfit [18,19,25,27]. To assess internal consistency of the factors, Cronbach’s alpha and omega were assessed and deemed acceptable if the values were ≥0.70 and ≤0.89 [18,19].

The same procedures and overall goodness of fit criteria utilized for the initial CFA were also used to evaluate acceptability of the covariance model of the identified EFA solution [23,25]. Localized areas of strain and modification indices were evaluated, and further refinement conducted if necessary.

#### 2.4.3. Multigroup Invariance Analysis

Multigroup invariance testing was conducted on the identified covariance solution across sex, history of mental health diagnosis, injury status, and activity level. For invariance analysis, physical activity levels were grouped as follows: Group 1 = inactive/low (i.e., inactive is defined as having no activity beyond baseline activity, and low activity is defined as having activity beyond baseline but fewer than 150 min of moderate intensity exercise per week); Group 2 = medium/high activity (i.e., medium is defined as having 150–300 min of moderate intensity activity per week, and high activity is defined as having more than 300 min of moderate intensity activity per week).

Invariance analysis evaluates measurement properties to ensure scoring on the instrument is not due to measurement error. Invariance procedures involved testing three different models: (1) configural, (2) metric, and (3) scalar [23]. The configural model assesses whether the model structure holds across subgroups. If the configural model holds, the more constrained metric model, which assesses the equivalency of factor loadings across subgroups, is tested. Finally, if the metric model holds, the scalar model is tested to assess item intercepts. To determine if the model is invariant, the configural model is evaluated using the same cutoff values described for the CFA and covariance model. The metric and scalar models are compared back to the configural model and considered invariant if the change in CFI (i.e., CFIdiff) is less than 0.01.

Invariant instruments indicate whether scores from the scale are outside of the measurement error [23], thus supporting the assessment of substantive questions (e.g., hypothesis testing for mean or variance differences). Thus, if measurement invariance was met, an equal variance and means model will be assessed using the same criteria during measurement testing (i.e., CFIdiff > 0.01). If the CFIdiff exceeds 0.01 compared to the configural model for either the latent variances or latent means, the groups are considered different on the tested metric (i.e., latent variances or means).

## 3. Results

### 3.1. Confirmatory Factor Analysis

**Figure 1:**
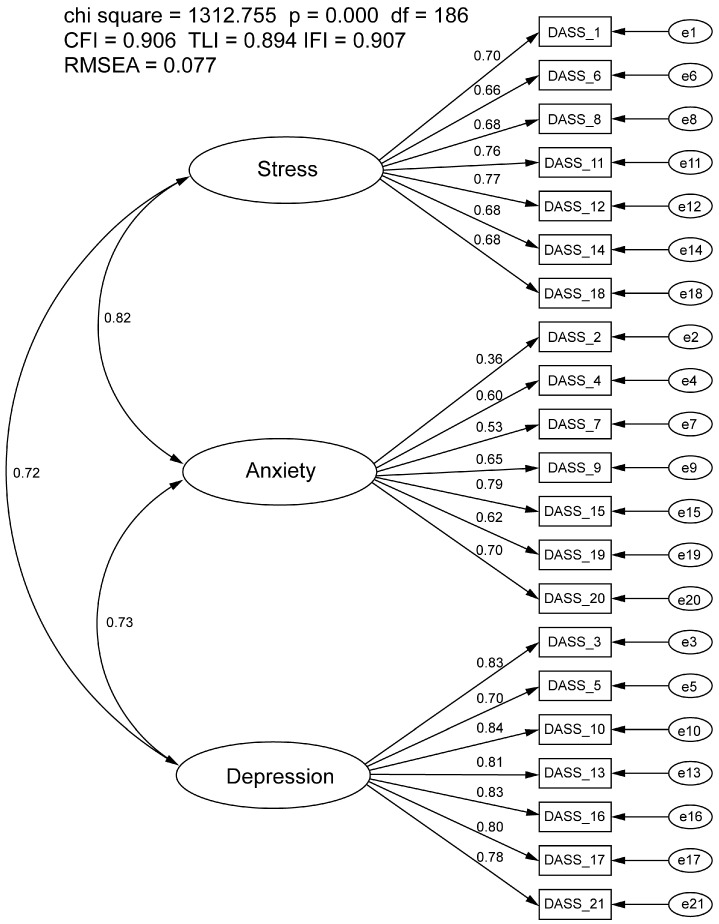
Confirmatory factor analysis of the Depression Anxiety Stress Scale-21. All factor loadings were significant at *p* < 0.001. Circle icons represent an unobserved variable (e.g., Stress = stress latent variable construct, e1 = error term 1). Rectangle icons represent observed variables (e.g., DASS_1 = survey item #1 on the DASS questionnaire).

The CFA of the three-factor, 21-item DASS approached but did not meet recommended goodness-of-fit indices (CFI = 0.906, TLI = 0.894, RMSEA = 0.077, IFI = 0.907; Figure 1) in the full sample. All factor loadings were significant, ranging from 0.36 to 0.84 and correlations between latent variables were significant but high (anxiety and stress r = 0.82; stress and depression r = 0.72; anxiety and depression r = 0.73). In addition to the high latent variable correlations, modification indices suggested that several meaningful cross-loadings were present. Therefore, an EFA was conducted using sample n1 on the DASS-21 to identify a more parsimonious model.

### 3.2. Exploratory Factor Analysis

The initial EFA of the DASS-21 using sample n1 (sex: females n = 404, 78.0%, males n = 112, 21.6%; history of mental health diagnosis: yes n = 232, 44.8%, no n = 275, 53.1%; injury status: healthy n = 322, 62.2%, injured n = 195, 37.7%; activity status: inactive/low n = 298, 57.7%, medium/high n = 220, 42.5%) extracted three factors with eigenvalues > 1.0 that accounted for at least 5% of the variance and 58.31% of the shared variance. Parallel analysis indicated that two factors should be retained; however, the eigenvalue for the third factor was slightly less than the random data eigenvalue: 1.24 and 1.31, respectively. Therefore, because the other three criteria outlined for factor retention were met (i.e., eigenvalues > 1.0, factors accounting for at least 5% of the variance, scree plot assessment ascertained that three factors was appropriate), three factors were retained. A total of ten items were removed due to low loadings, high cross-loadings, high interitem correlations, and inflated Cronbach’s alpha levels. The refined DASS (DASS-11) accounted for 69.17% of the variance with all factor loadings ≥ 0.42 (Table 2). Factor 1 contained three items from the original depression factor, factor 2 contained four items from the original stress factor, and factor 3 contained four items from the original anxiety factor. The DASS-11 was then tested in a covariance model using sample n2.

### 3.3. Covariance Model

**Figure 2:**
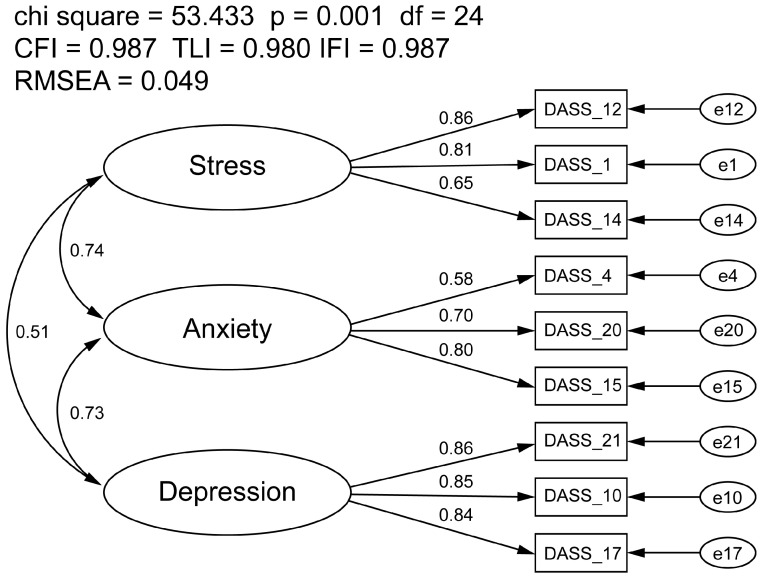
Covariance model of the Depression Anxiety Stress Scale-21. All factor loadings were significant at *p* < 0.001. Circle icons represent an unobserved variable (e.g., Stress = stress latent variable construct, e1 = error term 1). Rectangle icons represent observed variables (e.g., DASS_1 = survey item #1 on the DASS questionnaire).

The covariance model of the DASS-11 using sample n2 (sex: females n = 397, 76.8%, males n = 113, 21.9%; history of mental health diagnosis: yes n = 193, 37.3%, no n = 316, 61.1%; injury status: healthy n = 322, 62.2%, injured n = 196, 37.8%; activity status: inactive/low n = 285, 55.0%, medium/high n = 233, 45.0%) met some but not all recommended goodness-of-fit indices (CFI = 0.969, TLI = 0.953, RMSEA = 0.058, IFI = 0.970; Supplemental Figure S1). All factor loadings were significant, ranging from 0.62 to 0.87, and correlations between latent factors were moderate-to-high (range = 0.51 to 0.74). Further inspection of the modification indices revealed that there were items (#11 and #19) with meaningful cross-loadings, and each were subsequently removed. A final three-factor, nine-item scale (DASS-9) was retained that met all model fit indices (CFI = 0.987, TLI = 0.980, RMSEA = 0.049, IFI = 0.987; Figure 2). The shared variance between the three factors was 74.1%. All factor loadings were significant, ranging from 0.65 to 0.86, and correlations between latent factors were moderate-to-high (range = 0.51 to 0.74).

### 3.4. Invaraince Testing

#### 3.4.1. Sex

The DASS-9 models met all model fit criteria for males and females (Supplemental Table S2). The model was also invariant across sex, meeting all model fit criteria for the configural, metric, and scalar models, with a CFIdiff of <0.01 compared to the configural model. Because metric and scalar invariance was met, latent variances and means could be compared across subgroups, respectively: significant sex differences for equal variances and latent means models were not found across all factors.

#### 3.4.2. Mental Health Diagnosis

The models met all fit criteria for individuals with and without a previous or current mental health diagnosis (Supplemental Table S3); however, RMSEA values slightly exceeded the optimal threshold (≤0.06) for individuals without a mental health diagnosis (RMSEA = 0.062) but still fell within the acceptable range (≤0.08). The DASS-9 was also invariant across individuals with and without a previous or current mental health diagnosis. The configural, metric, and scalar models all met model fit criteria, supporting assessment of latent subgroup variances and means. Individuals with a history of a mental health diagnosis (i.e., previous or current) had higher variances and latent mean scores across anxiety, depression, and stress subscales, indicating a wider spread of scores and higher average scores across each dimension compared to individuals without a history of a mental health diagnosis.

#### 3.4.3. Injury Status

The DASS-9 models held for healthy and injured individuals, meeting all model fit criteria, except for RMSEA for healthy individuals. The RMSEA value (0.07) exceeded the optimal threshold (≤0.06) but still fell within the acceptable threshold (≤0.08; Supplemental Table S4). The DASS-9 model was invariant across injury status (i.e., healthy vs. injured), supporting latent variances and means testing; latent variances and means were not significantly different across injury status groups, indicating that both groups, regardless of injury status, had similar scores and a spread of scores across the depression, anxiety, and stress constructs.

#### 3.4.4. Activity Level

Model fit indices were met when assessing across activity levels (i.e., individuals who were inactive or had low levels of activity and for those with moderate-to-high levels of activity). RMSEA exceeded (0.076) the optimal cutoff value (≤0.06) for individuals with moderate-to-high activity levels but still fell within the acceptable range (≤0.08; Supplemental Table S5). The model was invariant across activity level, with model fit criteria met for configural, metric, and scalar models, supporting assessment of latent variances and means. Latent variances were equal across activity levels. However, those who were inactive or only had low levels of physical activity tended to have higher average anxiety, depression, and stress subscale scores.

## 4. Discussion

Currently, mental health disorders affect 1 in 5 adults in the United States [2]; therefore, there is a need to establish a psychometrically sound instrument that can serve research and clinical practice purposes. The DASS-21 is a frequently used instrument to assess psychological distress; however, previous psychometric studies of the DASS-21 demonstrated the model often failed to meet contemporary recommendations. For example, CFI values were considered satisfactory with outdated criteria of ≥0.90 [10,13,16,17,28,29] rather than the more contemporary value of ≥0.95 fit [24]. Additionally, invariance testing between subgroups of interest had not been completed. Therefore, further assessment of the DASS-21 in a large heterogenous sample was warranted.

Historically, the DASS-21 was believed to exhibit a three-factor, 21-item structure [9,12]. However, our CFA findings, consistent with other findings [28,30,31,32], indicate that the DASS-21 does not display sound factorial and structural validity, which warranted further testing to find a more parsimonious model. An exploratory factor analysis was conducted, and an 11-item model was identified. The 11-item model was then reduced to a 9-item model during covariance modeling due to problematic items, which indicated significant cross-loadings were present. The nine-item model included three distinct factors (i.e., depression, anxiety, stress), mirroring the original DASS-21 model. This finding is similar to previous research that has identified condensed models: 12-item model [31,32], 9-item model [32], and 8-item model [30]. However, the number and chosen items in each factor is different to previous findings; this may be due to different samples being used (e.g., clinical vs. healthy, different cultures, physically active vs. sedentary), different methodology (e.g., CTT), and/or decision making at the analysis level. It was outside the scope of the current study, but future research should assess and identify which model is optimal. Due to the discrepancies found for the DASS-21, future research should continue to assess its validity. Additionally, future research should assess the DASS-9 in a new sample to further establish validity (e.g., criterion), as well as the reliability, sensitivity, and specificity of the instrument.

Invariance testing ensures that differences found are outside of the measurement error and is a crucial step in further establishing validity [23]. The DASS-9 was invariant across sex, with males and females scoring similarly on the DASS-9, which aligns with prior findings on sex differences for the DASS-21 [33]. However, this contrasts with prior research where sex-based differences in certain mental health diagnoses were found [34]. For instance, women have a higher prevalence of mood and anxiety disorders and have more suicide attempts than men [34]. However, men tend to have lower levels of life satisfaction and are three times more likely to die by suicide [35,36]. It seems that both sexes experience psychological distress, as suggested by the DASS-9, but how it is experienced may be different, and further research should explore that nuance.

Typically, individuals with a history of mental illness score higher on the DASS-21 than those without a history [11], as do in-patient groups when compared to out-patient populations [37]. Our findings add to previous literature: the DASS-9 was invariant between individuals with and without a mental health diagnosis and can be used to assess group differences in this population; we found that individuals with a history of a mental health diagnosis scored higher compared to those without a mental health diagnosis. The findings serve as preliminary evidence for construct validity [27]; however, the DASS-9 should not be used as a diagnostic tool but rather as a screening tool.

The DASS-9 was invariant across injury status (i.e., those with an acute, subacute, chronic, or persistent injury, compared to those without an injury), which aligns with previous research [38] that also established invariance between injury status in the DASS-21. However, previous researchers found statistically significant differences in the DASS-21 between injured and noninjured individuals [38], which contrasts with our findings. This could potentially be due to the different DASS models used (i.e., 21-item vs. 9-item) or the different subsamples surveyed (e.g., athletic vs. nonathletic samples) in each study. An association between musculoskeletal pain and psychological concerns [5] has been demonstrated. For example, higher levels of stress, depressed mood states, and anxiety have all been associated with higher injury occurrence [6]. Therefore, future research should seek to collect responses to the DASS-9 from injured and healthy individuals to understand how psychological distress is being experienced by these two subgroups.

Invariance was identified across different activity levels (i.e., inactive/low vs. moderate/high). Several studies have demonstrated a relationship between physical activity, depression, anxiety, and stress [39,40,41,42,43], with evidence that exercise can be used to treat symptoms of mild depression and reduce anxiety levels [40]. Similarly, we found that individuals who were inactive or participated in low levels of activity had higher levels of anxiety, depression, and stress compared to those who participated in moderate-to-high levels of activity. Additionally, a study that used the DASS-21, found that those with low levels of physical activity (i.e., sedentary lifestyle) had higher scores/rates of depression, anxiety, and stress compared to those who fell into the stratifications of irregularly active, active, and very active [44]. Therefore, our study further adds to the body of literature and provides preliminary evidence that the DASS-9 can adequately detect and assess mean differences between these groups.

### Limitations and Future Research

Our study, despite its large sample size, was limited by a lack of diversity in the sample, with most participants identifying as female (77%) and white ethnicity (85%). Future research should aim to incorporate a more heterogeneous sample. Additionally, while we assessed invariance across several key groups, we were unable to evaluate invariance between age groups due to limitations in sample size and grouping. Future research should aim to address invariance testing for age groups to ensure broader applicability. Although we identified a condensed version of the DASS, further data collection with a new, representative sample is needed to fully validate its structural integrity [23]. Additionally, despite identifying a psychometrically sound factor structure for the DASS (i.e., DASS-9), a proliferation of condensed DASS versions in the literature may cause confusion for researchers and clinicians who have to determine which version to use. Thus, future research should compare the condensed DASS instruments (i.e., 8 items, 9-items, and 11-items) to make a more substantiated recommendation for practical use across these instruments. Notably, we advise against using the DASS-9 as a diagnostic tool or for treatment decisions until further validation is conducted.

## 5. Conclusions

The original three-factor DASS-21 failed to meet recommended contemporary model fit criteria; therefore, it is not recommended for use. We identified a refined three-factor, nine-item scale (DASS-9) that met recommended fit indices and may be a more viable option to use for assessing psychological distress. Our findings also present preliminary evidence that the DASS-9 can be used for group comparison across sex, mental health diagnosis, injury status, and physical activity status, with significant differences found between mental health diagnosis groups and activity status groups. Notably, higher levels of anxiety, depression, and stress were reported for individuals with a history of mental health diagnosis and those that were inactive or participated in low levels of activity. While this study contributes to the literature by identifying a more efficient tool for assessing psychological distress, while preserving construct validity, it is important to note that the DASS-9 should only be used as a screening tool and not as a diagnostic measure. Further research is recommended prior to widespread adoption to compare the DASS-9 to other condensed versions and to examine measurement properties across repeated use (i.e., time) of the scale in more diverse populations.

## Figures and Tables

**Table 1:** Demographics. Estimated characteristics of participants.

Characteristic	N (%)
Sex	
Male	225 (21.7)
Female	801 (77.3)
Unknown	10 (1.0)
Education	
High school or GED	34 (3.3)
Some college, no degree	125 (12.1)
Associate degree	58 (5.6)
Bachelor’s degree	299 (28.9)
Master’s degree	355 (34.3)
Doctoral degree	138 (13.3)
Other	25 (2.4)
Unknown	2 (0.2)
Mental Health Diagnosis	
Yes	425 (41.0)
No	591 (57.0)
Unknown	20 (1.9)
Ethnicity	
Caucasian	883 (85.2)
African American	51 (4.9)
Hispanic	60 (5.8)
Other	25 (2.4)
Unknown	17 (1.6)
Activity Level	
Inactive	180 (17.4)
Low	403 (38.9)
Medium	332 (32.0)
High	121 (11.7)
Injury Status	
Healthy	645 (62.2)
Acute injury	20 (1.9)
Subacute injury	26 (2.5)
Persistent injury	108 (10.4)
Chronic injury	237 (22.9)

**Table 2:** Exploratory factor analysis DASS-11. Exploratory factor analysis results of the refined DASS-11.

Item	1	2	3
21. I felt that life was meaningless	0.901		
10. I felt that I had nothing to look forward to	0.847		
17. I felt I wasn’t worth much as a person	0.842		
12. I found it difficult to relax		0.877	
1. I found it hard to wind down		0.840	
14. I was intolerant of anything that kept me from getting on with what I was doing		0.494	
11. I found myself getting agitated		0.475	
19. I was aware of the action of my heart in the absence of physical exertion (e.g., sense of heart rate increase, heart missing a beat)			0.805
4. I experienced breathing difficulty (e.g., excessively rapid breathing, breathlessness in the absence of physical exertion)			0.666
20. I felt scared without any good reason			0.493
15. I felt I was close to panic			0.419
Eigenvalue	5.19	1.33	1.10
Cronbach’s alpha	0.89	0.82	0.77
Omega	0.89	0.83	0.78

## Data Availability

Data can be made available upon reasonable request.

## Supplementary Materials

The following supporting information can be downloaded at: https://www.mdpi.com/article/10.3390/ejihpe14110192/s1, Supplemental Table S1: Study Definitions and Terminology. Supplemental Tables S2–S5: Invariance Testing between Groups. Supplemental Figure S1: Covariance Model of the DASS-11.
