# Supplementary material for: Examining the Factor Structure and Validity of the Depression Anxiety Stress Scale-21
Source: Eur J Investig Health Psychol Educ. 2024 Nov 20;14(11):2932–43. doi: 10.3390/ejihpe14110192 (PMC11592842; doi:10.3390/ejihpe14110192)

Supplemental Tables and Figure

Supplemental Table S1. Study Definitions and Terminology.

**Terminology**	**Definition**
**Injury Status Classification**	
Healthy	"Free from musculoskeletal injury and fully able to participate in sport or activity."
Acute Injury	"A musculoskeletal injury that precludes full participation in sport or activity for at least 2 consecutive days (0-72 hours post-injury)."
Subacute Injury	"A musculoskeletal injury that precludes full participation in sport or activity for at least 2 consecutive days (3 days to 1-month post-injury)."
Persistent Injury	"A musculoskeletal injury that has been symptomatic for at least 1 month."
Chronic Injury	"Pain that consistently does not get any better with routine treatment or non-narcotic medication."
**Activity Level Status Classification**	
Inactive	"No activity beyond baseline activity (baseline activity refers to 'light-intensity activities [e.g., standing, walking, lifting weighted objects] of daily life)."
Low	"Activity beyond baseline, but fewer than 150 minutes of moderate intensity exercise per week (moderate activity includes activities such as brisk walking, yoga, lifting weights, etc.)."
Medium	"150-300 minutes of moderate intensity activity per week".
High	"More than 300 minutes of moderate intensity activity per week."

Supplemental Table S2. Goodness-of-fit indices for measurement invariance analyses between sex.

	χ^2	df	χ^2_{diff} (df_{diff})	CFI	CFI_{diff}	TLI	RMSEA
Male (n = 225)	42.168	24	----	.986	----	.979	.058
Female (n = 801)	84.11	24	----	.981	----	.972	.056
Configural Model (equal form)	126.33	48	----	.983	----	.974	.040
Metric Model (equal loadings)	132.00	54	3.67 (6)	.983	NC	.977	.038
Equal Factor Variances Model	136.23	57	9.9 (9)	.982	.001	.978	.037
Scalar Model (equal indicator intercepts)	142.79	60	16.43 (12)	.982	.001	.978	.037
Equal Latent Means Model	161.49	63	35.16 (15)	.978	.005	.975	.039

Supplemental Table S3. Goodness-of-fit indices for measurement invariance analyses between mental health diagnoses.

	χ^2	df	χ^2_{diff} (df_{diff})	CFI	CFI_{diff}	TLI	RMSEA
Mental health diagnosis (n = 425)	38.28	24	----	.992	----	.988	.037
No mental health diagnosis (n = 591)	77.71	24	----	.975	----	.962	.062
Configural (equal form)	115.97	48	----	.983	----	.974	.037
Metric Model (equal loadings)	126.13	54	10.16 (6)	.982	.001	.976	.036
Equal Factor Variance Model	227.79	57	111.82 (9)	.957	.026	.946	.054
Scalar Model (equal indicator intercepts)	141.37	60	29.32 (12)	.980	.003	.976	.037
Equal Latent Means Model	227.40	63	111.43 (15)	.959	.024	.953	.051

Supplemental Table S4. Goodness-of-fit indices for measurement invariance analyses across injury status.

	χ^2	df	χ^2_{diff} (df_{diff})	CFI	CFI_{diff}	TLI	RMSEA
Healthy (n = 644)	99.53	24	----	.972	----	.959	.070
Injured (n = 391)	33.44	24	----	.994	----	.991	.032
Configural (equal form)	132.95	48	----	.981	----	.971	.041
Metric Model (equal loadings)	138.21	54	5.26 (6)	.981	NC	.974	.039
Equal Factor Variance Model	151.73	57	18.78 (9)	.978	.003	.973	.040
Scalar Model (equal indicator intercepts)	156.60	60	23.65 (12)	.977	.004	.973	.040
Equal Latent Means Model	185.14	63	52.19 (15)	.972	.009	.968	.043

NC = no change

Supplemental Table S5. Goodness-of-fit indices for measurement invariance analyses across activity level.

	χ^2	df	χ^2_{diff} (df_{diff})	CFI	CFI_{diff}	TLI	RMSEA
Inactive/Low (n = 583)	49.94	24	----	.989	----	.984	.043
Moderate/High (n = 453)	87.40	24	----	.969	----	.969	.076
Configural (equal form)	137.34	48	----	.980	----	.969	.042
Metric Model (equal loadings)	150.93	54	13.59 (6)	.978	.002	.971	.042
Equal Factor Variance Model	168.36	57	31.02 (9)	.975	.005	.968	.043
Scalar Model (equal indicator intercepts)	176.72	60	39.38 (12)	.973	.007	.968	.043
Equal Latent Means Model	215.68	63	78.34 (15)	.965	.015	.960	.048

Supplemental Figure S1. Covariance Model of the DASS-11.

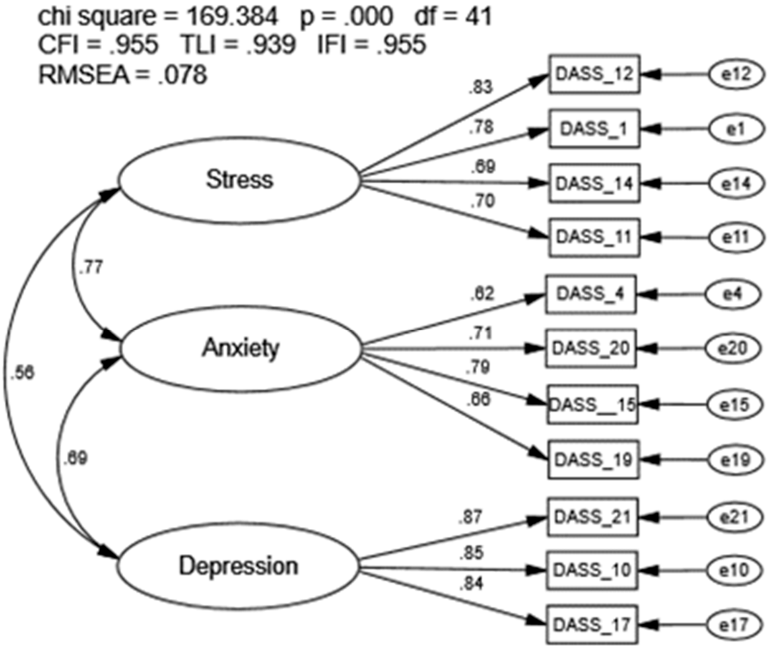

Supplement: Supplementary file 1 [file ejihpe-14-00192-s001.zip › ejihpe-3229934-supplementary.pdf]
